# A Comprehensive Review of Gene Mutations in Inherited Blood Disorders Among the Saudi Population

**DOI:** 10.1155/humu/2418005

**Published:** 2026-04-01

**Authors:** Nancy S. Younis, Rahma M. Alkabsh, Shahad M. Nasser Alqahtani, Hajer Aljuail, Manar A. Alhashim, Shahad A. Bokhamsin, Layla J. Albaqshi, Salsabil F. Alqadhib, Jumanah A. Aldandan, Zahra A. Alshakhs, Maryam H. Altaweel, Maged E. Mohamed

**Affiliations:** ^1^ Department of Pharmaceutical Sciences, College of Clinical Pharmacy, King Faisal University, Al-Ahsa, Saudi Arabia, kfu.edu.sa

**Keywords:** bleeding gene mutation, genetic counseling, inherited blood disorders

## Abstract

**Background:**

Inherited blood disorders (IBDs) are a major health concern in the Kingdom of Saudi Arabia (KSA), largely due to the high prevalence of consanguineous marriages.

**Objectives:**

This review is aimed at summarizing gene mutations and variants associated with IBDs in the Saudi population to enhance diagnosis and personalized care.

**Methods:**

Published studies on IBD‐related genetic mutations in Saudis were systematically retrieved from PubMed, Web of Science, Google Scholar, and EGEMS database using keywords “gene,” “Saudi,” “polymorphism,” and “the different inherited blood disorders.” A total of 118 studies published between 2015 and 2024 met the inclusion criteria.

**Results:**

The *β*‐globin (*HBB*) gene showed the greatest mutational diversity, with over 60 *β*‐thalassemia variants identified. The *α*‐globin genes (*HBA1*, *HBA2*, and the unique *HBA12*) were frequently involved in *α*‐thalassemia, with the –*α3.7* deletion predominating. In sickle cell disease, the *HbS* mutation (*c.20A*>*T*) is the most common, primarily linked to the Arab–Indian haplotype, whereas polymorphisms in *BCL11A*, *HBS1L-MYB*, and *ANTXR1* influenced fetal hemoglobin levels. Frequent thrombophilia‐related variants occurred in *F5*, *SERPINC1*, *MTHFR*, and *FII*, and inherited thrombocytopenias were linked to *MPL*, *ANKRD26*, *THPO*, *DIAPH1*, and *ADAMTS13*. Rare disorders such as Wiskott–Aldrich syndrome (WAS) and coagulation factor deficiencies (e.g., FX, F7, and F8) were also reported.

**Conclusion:**

The Saudi population exhibits a distinct and diverse spectrum of IBD‐related mutations. Understanding these genetic patterns can enhance diagnostic precision, guide genetic counseling, and advance personalized medicine initiatives across the Kingdom.

## 1. Introduction

Inherited blood disorders (IBDs) are identified as blood‐related conditions that are passed from parents to their offspring [1]. The Kingdom of Saudi Arabia (KSA) is experiencing a distinctive prevalence of IBDs [2]. For instance, the reported cases of Glanzmann′s thrombasthenia (GT) are fivefold higher in KSA than the global prevalence [3].

Since the genetic aspect is central to many blood disorders, consanguinity amplifies the incidence of IBDs among the Saudis. The rate of consanguineous marriages reaches up to 57.7% in some areas and may exceed 80% in rural regions [4]. The Saudi National Transformation Program, launched in 2016 by the KSA government, aims to ensure the realization of Vision 2030; a critical health feature of this program is to guarantee prevention as well as early intervention. Strategies to tackle the rise of IBDs included several initiatives [2]. A royal decree was issued in 2003 mandating premarital screening tests followed by hereditary counseling [5]. The screening program offers several benefits, including reducing future complications, enabling early detection, and facilitating risk assessment. As a result, the premarital screening program and the genetic counseling program substantially diminished at‐risk marriage by 60% [6].

Additionally, the Kingdom has promoted genetics information within the community via healthcare staff and primary healthcare centers and the media. Currently, the public appears to be more aware of the risks associated with consanguineous marriages. Up to date, the premarital hematological screening is done by biochemical testing to genetics testing. Due to the high rates of consanguinity, the Saudi population exhibits a unique genetic landscape, making whole‐exome sequencing (WES) a powerful tool for identifying novel variants and disease‐causing mutations. Using WES is critically important for uncovering the genetic basis of both rare and common diseases prevalent in the region [7].

Despite increased awareness of genetic illnesses, the persistently high rate of consanguineous marriages highlights the challenges posed by cultural marital practices. Therefore, understanding the epidemiology and grounds of inherited blood conditions is imperative as it will boost the therapeutic intervention, prompt early detection, better resource management, which will improve the quality of life for Saudis. Additionally, greater governmental efforts are required to promote alternative reproductive choices, launch new clinical protocols, and expand existing genetic screening programs.

The current review summarizes the key funding from the available literature on IBDs in KSA. This article enumerated all the described alterations associated with IBDs and merges them in a paper. These findings may ease additional national‐level inquiries to generate a nationwide repository of all blood diseases gene differences documented among Saudis.

## 2. Methods

### 2.1. Literature Search Strategy

IBDs were identified using international databases such as Online Mendelian Inheritance in Man (OMIM), National Organization for Rare Disorders (NORD), and the World Health Organization (WHO). These were cross‐referenced with national data from the Saudi Ministry of Health, Saudi Health Council registries, and King Faisal Specialist Hospital & Research Centre databases to ensure local clinical relevance. Electronic literature databases (PubMed, Scopus, Web of Science, Saudi Digital Library (SDL), and Google Scholar) were systematically searched for studies published between January 2015 and December 2024, aligning with the launch of the Saudi National Mental Health Survey (SNMHS) and ensuring the inclusion of recent, relevant data.

The search strategy combined disorder‐specific terms, genetic terminology, and population identifiers using Boolean operators as follows:

(“Inherited blood disorder” OR “Hemophilia” OR “Thalassemia” OR “Sickle cell disease” OR “Glanzmann Thrombasthenia” OR “Thrombophilia” OR “Wiskott–Aldrich syndrome”) AND (“mutation” OR “gene variant” OR “polymorphism” OR “genotype”) AND (“Saudi” OR “Kingdom of Saudi Arabia” OR “KSA”).

Searches were restricted to English‐language studies conducted on Saudi populations, and duplicates were removed before screening.

#### 2.1.1. Eligibility Criteria

Inclusion criteria included peer‐reviewed articles, case reports, and systematic reviews/meta‐analyses published in English between 2015 and 2024. Studies explicitly reporting genetic variants or polymorphisms among individuals of Saudi nationality

Exclusion criteria included non‐Saudi or mixed‐population studies without clear subgroup data, non‐English publications, and articles lacking methodological clarity, adequate sample size, or primary genetic data.

#### 2.1.2. Articles Screening and Selection

**Figure 1:**
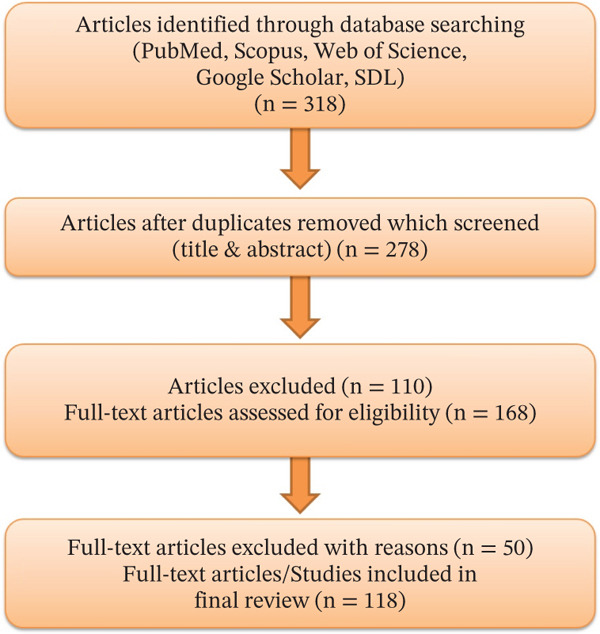
PRISMA flow diagram summarizing the article identification, screening and eligibility, and intabclusion process.

Titles and abstracts were first screened to identify studies explicitly conducted in Saudi Arabia as an initial screening. Full texts of potentially relevant articles were reviewed against inclusion/exclusion criteria. A PRISMA compliant flow diagram (Figure 1) illustrates the selection process. Out of 318 initially identified records, 256 remained after duplicate removal. Following title/abstract screening and full‐text eligibility assessment, 118 studies met all inclusion criteria and were included in the final review.

### 2.2. Data Extraction

From each eligible study, data were systematically extracted into a structured Excel sheet covering bibliographic details (authors, year, and journal), study design and sample characteristics (size, demographics, and region in KSA), investigated genes or polymorphisms, key findings, pathogenic or polymorphic variants, and reported clinical correlations. This structured approach ensured uniformity and reproducibility in data synthesis across all selected studies.

## 3. IBDs

### 3.1. Amyloidosis

Familial transthyretin amyloidosis (ATTR) is associated with increased formation of amyloid fibrils, which affect the peripheral nervous system and the heart. Transthyretin (*TTR*) gene prevents the protein from dissociating into monomers that can combine to form amyloid fibrils [8]. Abouelhoda et al. [9] analyzed 13,906 Saudi exomes and acknowledged three known and three novel, at the time, *TTR* mutations. The known variants were *c.239C*>*T* (*p.Thr80Ile*), *c.238C*>*G* (*p.Thr80Ala*), and *c.424G*>*A (p.Val142Ile*, most common), whereas the novel variants were *c.404C*>*T* (*p.Ser135Phe*), *c.428C*>*T* (*p.Thr143Ile*), and *c.298A*>*G* (*p.Lys100Glu*).

### 3.2. Bernard–Soulier Syndrome (BSS)

BSS is caused by the absence of glycoprotein GPIb‐V‐IX complex, von Willebrand factor (VWF) receptor, and clinically characterized by thrombocytopenia, giant platelets, and bleeding tendency [10]. Reported cases include a 7‐year‐old girl with a homozygous deletion of 39 nucleotides in Exon 2 of the glycoprotein Ib platelet subunit alpha (*GP1BA*) gene [11]. Another case described an 8‐year‐old boy with a homozygous nucleotide substitution (TTT‐TCT) resulting in Phe71Ser mutation in *glycoprotein IX* (*GPIX*) resulting in the lack of *GPIX* expression. A third study identified a homozygous glycoprotein Ib platelet subunit beta (*GP1BB*) variant *c.423C*>*A* (*p.Cys141Ter*), causing a premature stop codon in three members of the same family [12].

### 3.3. Congenital Nonspherocytic Anemia

This condition is caused by alterations in the pyruvate kinase L/R (*PKLR*). WGS of a female patient revealed a rare missense variant in the *PKLR* gene *c.1015G*>*A* (*p.D339N*) in a homozygous state [13].

### 3.4. Fanconi Anemia (FA)

FA is produced by gene mutations implicated in DNA damage response and repair [14]. Two cases were reported; a homozygous splice site variant *c.2605+1G*>*A* mutation in the FA complementation Group D2 (*FANCD2)* gene manifested as a severe ambiguous genitalia in a male infant [15] and a novel homozygous missense variant, c.3296C>G (p.Thr1099Arg) in *FANCN* gene, causing FA in a preterm female baby [16].

### 3.5. GT

GT is caused by mutations in integrin subunit alpha 2b (*ITGA2B*) and integrin subunit beta 3 (*ITGB3*) genes that code for the alpha and beta subunits of GPIIb/IIIa (*α*IIb*β*3), respectively, which impair platelet aggregation [17]. The prevalence of GT in Saudi Arabia is notably high. In Al‐Madinah, the estimated prevalence in 2022 was 1 in 10,000, significantly higher than the global average [18]. Table 1 summarizes all the reported *ITGA2B* and *ITGB3* variants associated with GT cases in KSA.

**Table 1:** All the reported *ITGA2B* and *ITGB3* variants associated with GT cases in Saudi Arabia showing mutations/variants reported (HGVS Expression), protein change, dbSNP/ClinVar IDs, GRCh38 coordinates, and the reported pathogenicity.

Source (ref)	No of cases	Gene	Mutations/variants reported (HGVS expression)	Protein change	dbSNP/ClinVar IDs	GRCh38 coordinates (Chr 17)	Reported pathogenicity
[19]	Six GT cases	*ITGB3*	NM_000212.3:c.2190delC	NP_000203.2:p.Ser703fs (p.Ser703fsX2)	VCV001210208.2	47,306,664	Pathogenic

[3]	72 cases	*ITGA2B*	NM_000419.5:c.1879‐2A>G		VCV000002904.3	44,379,481	Pathogenic.
			NM_000419.5:c.1651C>T	NP_000410.2:p.Arg551Trp	VCV000952955.1	44,380,488	Likely pathogenic or pathogenic
			NM_000419.5:c.1616T>G	NP_000410.2:p.Leu539Arg	VCV000953057.7	44,380,523	Likely pathogenic or pathogenic
			NM_000419.5:c.985G>T	NP_000410.2:p.Val329Phe	VCV000952955.1	44,383,907	Likely pathogenic
			NM_000419.5:c.558C>G	NP_000410.2:p.Tyr186∗ (or p.Tyr186Ter)	rs1416238665/VCV000953014.2	44,385,837	Pathogenic
			NM_000419.5:c.1210+5G>A			44,383,490	VUS
			NM_000419.5:c.1620G>C	NP_000410.2:p.Gln540His	VCV000953057.7	44,380,519	Likely pathogenic or pathogenic
			NM_000419.5:c.1142C>T	NP_000410.2:p.Thr381Ile	VCV001332427.1	44,383,763	Likely pathogenic or pathogenic
		*ITGB3*	NM_000212.3:c.2302‐1G>A	NP_000203.2:p.? (splice variant)	VCV000859345.2	47,305,572	Pathogenic
			NM_000212.3:c.2112delC	NP_000203.2:p.Leu705Cysfs∗4	VCV000953061.1	47,306,583	Pathogenic
			NM_000212.3:c.727G>C	NP_000203.2:p.Asp243His	VCV000859317.1	47,314,642	Pathogenic
			NM_000212.3:c.1265G>A	NP_000203.2:p.Ser422Asn	None assigned	47,310,230	Pathogenic
			NM_000212.3:c.662C>T	NP_000203.2:p.Thr221Met	rs1556061327	47,314,707	Pathogenic
			NM_000212.3:c.985A>G	NP_000203.2:p.Asn329Asp	rs397508493	47,313,444	Pathogenic
			NM_000212.3:c.1835G>A	NP_000203.2:p.Cys612Tyr	rs758564032	47,307,434	Pathogenic
			NM_000212.3:c.1539delC	NP_000203.2:p.Ser513fs	VCV000624021.1	47,308,300	Pathogenic
			NM_000212.3:c.437T>C	NP_000203.2:p.Leu146Pro	None assigned	47,316,211	Pathogenic

[20]	2‐year‐old GT	*ITGA2B*	NM_000419.5:c.985G>T	NP_000410.2:p.Val329Phe	RCV001332427.1	44,383,90	Likely pathogenic or pathogenic
[21]	Nine GT cases	*ITGB3*	NM_000212.3:c.2113delC	NP_000203.2:p.Leu705Cysfs∗4	VCV000953061.1	47,306,582	Pathogenic

[18]	125 GT cases	*ITGA2B*	NM_000419.5:c.1879‐2A>G	NP_000410.2:p.? (splice variant)	VCV000002904.3	44,379,481	Pathogenic
		*ITGB3*	NM_000212.3:c.1210+5G>A	NP_000203.2:p.? (splice variant)	None assigned	~47,311,700 (approx.)	VUS
[22]	One GTLS case	*ITGB3*	NM_000212.3:c.1123G>A	NP_000203.2:p.Gly375Arg	rs104894297/VCV000018593.1	47,312,246	Likely pathogenic or pathogenic

Abbreviations: GT, Glanzmann thrombasthenia; GTLS, Glanzmann thrombasthenia‐like syndrome; *ITGA2B*, integrin subunit alpha 2b; *ITGB3*, integrin subunit beta 3; VUS, variant of uncertain significance.

### 3.6. Hemophagocytic Lymphohistiocytosis (HLH)

Primary HLH is produced by hereditary alterations of the *FLH* loci mainly among other genes. Secondary HLH occurs with infections, malignancy, autoimmune diseases, and certain metabolic disproportions [23]. Elyamany et al. [24] reported the first case series of 12 HLH Saudi cases; nine were primary HLH and 3 were secondary HLH patients. Genetic mutational investigation exposed syntaxin‐11 (*STX11*) mutation in five cases and perforin‐1 (*PRF1*) mutation in two cases.

### 3.7. Hemophilia

Hemophilias are a group of X‐linked genetic conditions presented with an absence in blood coagulation factors. Hemophilia A (HA) is associated with mutations in Factor VIII (*FVIII*) genes, Hemophilia B (HB) resulted from mutations in Factor *IX* gene (*FIX*), and Hemophilia C (HC) is due to mutations in Factor *XI* genes (*FXI*) [25]. Al‐Allaf, F.A. et al. [25] appraised that as a minimum of 1000 Saudis are probable influenced by HA and HB. Unfortunately, there are not many information about the nature of shared variants triggering HA or HB.

#### 3.7.1. HA

Table 2 presents the spectrum of *FVIII* gene mutations recognized in HA patients including missense, nonsense, frameshift, and splicing variants.

**Table 2:** The reported gene variants of FVIII reported with HA patients and the reported gene variants associated with IDA in the Saudi population.

Source (ref)	No of cases	Gene	Mutations/variants reported (HGVS expression)	Protein change	dbSNP/ClinVar IDs	GRCh38 coordinates (Chr X)	Reported pathogenicity
**Hemophilia A (HA)**							
[26]	Six HA cases	*FVIII*	NM_000132.4:c.5194A>G	NP_000123.1:p.Lys1732Arg	rs121964868/VCV000186178.5	154,924,960	Likely patho or pathogenic
			NM_000132.4:c.352A>C	NP_000123.1:p.Thr118Pro		154,986,527	Pathogenic
			NM_000132.4:c.1963G>T	NP_000123.1:p.Gly655∗		154,957,002	Pathogenic
			NM_000132.4:c.866C>T	NP_000123.1:p.Ser289Leu	VCV000589856.5	154,976,559	Likely pathogenic or pathogenic

[27]	22 HA cases		NM_000132.4:c.409A>C	NP_000123.1:p.Thr137Pro		~154,986,470 (approx.)	Pathogenic
			NM_000132.4:c.760A>G	NP_000123.1:p.Asn254Asp	VCV000869151.1	~154,979,530 (approx.)	Pathogenic
			NM_000132.4:c.923C>T	NP_000123.1:p.Ser308Leu		~154,976,400	Likely pathogenic or pathogenic
			NM_000132.4:c.1836G>C	NP_000123.1:p.Arg612Pro		~154,959,700 (approx.)	Pathogenic
			NM_000132.4:c.5194A>G	NP_000123.1:p.Lys1732Arg	rs121964868/VCV000186178.5	154,924,960	Pathogenic
			NM_000132.4:c.3735_3744delins	NP_000123.1:p.Leu1246fs		~154,941,600	Pathogenic

[28]	110 HA cases		NM_000132.4:c.355G>C	NP_000123.1:p.Ala119Pro		~154,986,524	Pathogenic
			NM_000132.4:c.6482delC	NP_000123.1:p.Pro2161Leufs∗25	VCV000527376.1	~154,917,998	Pathogenic
			NM_000132.4:c.409A>C	NP_000123.1:p.Thr137Pro		~154,986,470	Pathogenic
			NM_000132.4:c.1804C>T	NP_000123.1:p.Arg602∗	rs104894451, VCV000018610.6	154,959,731	Pathogenic

[29]	26 HA Saudi Patients		Del Exons 8–14	Absent/truncated protein		~154.9 Mb region	Pathogenic
			Del Exons 7–10	Absent/truncated protein		~154.9 Mb region	Pathogenic
			Del Exon 4	Absent/truncated protein		~154.9 Mb region	Pathogenic
			NM_000132.4:c.1021G>C	NP_000123.1:p.Ala341Pro		~154,975,400	Pathogenic
			NM_000132.4:c.1183A>C	NP_000123.1:p.Lys395Gln		~154,973,800	Pathogenic
			NM_000132.4:c.1930T>A	NP_000123.1:p.Leu644Met		~154,957,100	Pathogenic
			NM_000132.4:c.6322G>C	NP_000123.1:p.Ala2108Pro	VCV000527375.1	~154,918,100	Pathogenic

[25]	21 HA cases		NM_000132.4:c.99G>T	NP_000123.1:p.Trp33Cys	rs104894458, VCV000018619.4	154,987,620	Pathogenic
			NM_000132.4:c.2138delA	NP_000123.1:p.Asn713Thrfs∗9	VCV000859265.1	154,954,586	Pathogenic
			NM_000132.4:c.6430G>A	NP_000123.1:p.Glu2144Lys		~154,918,050	Likely pathogenic or VUS
			NM_000132.4:c.760A>G	NP_000123.1:p.Asn254Asp	VCV000869151.1	~154,979,530	Pathogenic
			NM_000132.4:c.1804C>T	NP_000123.1:p.Arg602∗	rs104894451/VCV000018610.6	154,959,731	Pathogenic
			NM_000132.4:c.1836G>C	NP_000123.1:p.Arg612Pro	None assigned	~154,959,700	Pathogenic

[30]	25 Saudi female		NM_000132.4:c.2507C>G	NP_000123.1:p.Ser836Pro	rs1345691509	~154,950,550	Likely pathogenic or pathogenic
					NM_000132.4:c.3338T>G	NP_000123.1:p.Met1113Thr	rs781800942

[31]	96 HA patients		NM_000132.4:c.5816‐2A>G	NP_000123.1:p.? (splice variant)	rs2123996454	~154,924,400	Pathogenic
			g.164496G>A (intron/exon 23)	NP_000123.1:p.? (splice variant)		164,496 (genomic coordinate in Intron 22)	Pathogenic
			NM_000132.4:c.409A>C	NP_000123.1:p.Thr137Pro		~154,986,470	Pathogenic
			NM_000132.4:c.760A>G	NP_000123.1:p.Asn254Asp	VCV000869151.1	~154,979,530	Pathogenic
			NM_000132.4:c.1835G>C	NP_000123.1:p.Arg612Pro		~154,959,700	Pathogenic

[32]	21 patients, negative for inv‐1/inv‐22,		NM_000132.4:c.6130_6131insC	NP_000123.1:p.Leu2044Profs∗9	VCV000527373.1	~154,920,800	Pathogenic
			NM_000132.4:c.5815G>C	NP_000123.1:p.Ala1939Pro		~154,924,400	Pathogenic
			NM_000132.4:c.5493C>G	NP_000123.1:p.Thr1831Thr	rs758784381	~154,924,800	VUS
			NM_000132.4:c.3734_3740delinsATTTCT	NP_000123.1:p.Leu1245fs		154,917.900	VUS
			NM_000132.4:c.6545G>A	NP_000123.1:p.Arg2182His	rs121964872/VCV000018625.6	154,917,900	Pathogenic
			NM_000132.4:c.6107A>G	NP_000123.1:p.Tyr2036Cys		~154,920,800	Pathogenic
			NM_000132.4:c.1063C>T	NP_000123.1:p.Arg355∗	rs104894459/VCV000018620.4	154,975,100	Pathogenic

**Hemophilia B (HB)**							

[25	42 DNA samples	*FIX*	NM_000133.5:c.855G>C	NP_000124.2:p.Glu285Asp		~154,611,600	Pathogenic
			NM_000133.5:c.316G>A	NP_000124.2:p.Gly106Ser	rs104894042/VCV000018512.4	154,625,979	Pathogenic
			NM_000133.5:c.580G>A	NP_000124.2:p.Ala194Thr	rs104894047/VCV000018517.3	154,614,845	Pathogenic
			NM_000133.5:c.1136G>A	NP_000124.2:p.Arg379Gln	rs104894060/VCV000018529.4	154,606,536	Pathogenic
			NM_000133.5:c.1025C>T	NP_000124.2:p.Thr342Met	rs104894059/VCV000018528.3	154,608,131	Pathogenic
[31]	39 HB Saudi patients		NM_000133.5:c.580G>A	NP_000124.2:p.Ala194Thr	rs104894047/VCV000018517.3	154,614,845	Pathogenic
					NM_000133.5:c.880C>T	NP_000124.2:p.Arg294∗	rs104894056/VCV000018525.4

**Combined Factors V and VIII deficiency (F5F8D)**							

[33]	A 7‐year‐old boy	*LMAN1*	NM_005570.4:c.822G>A	NP_005561.1:p.Glu274=		Chr 18: 59,348,799	Pathogenic

**3.9 Iron deficiency anemia (IDA)**							

[34]	—	*TF*	NM_001002052.2:c.1596+372A>G	Intronic (no direct protein change)	rs3811647	Chr 3: 50,885,824	Not classified as pathogenic
[35]	108 female in northern KSA	*TMPRSS6*	NM_144686.3:c.1906A>G	NP_659809.2:p.Ala636Val	rs855791	Chr 2: 166,419,058	Not classified as Pathogenic
			NM_144686.3:c.905+140G>A	Intronic (no direct protein change)	rs2111833	Chr 2: 166,427,330	
[36]	108 Saudi female	*TMPRSS6*	NM_144686.3:c.849‐55 T > C	Intronic (no direct protein change)	rs1421312	Chr 2: 166,427,458	Increased risk of IDA
		*BMP2*	NM_001200.3:c.1143+1561T>C		rs235756	Chr 20: 54,411,357	
[37]	Six cases with IRIDA	*TMPRSS6*	NM_144686.3:c.218G>A	NP_659809.2:p.Trp73∗		Chr 2: ~166,450,200	Pathogenic
			NM_144686.3:c.2207T>C	NP_659809.2:p.Val736Ala		Chr 2: ~166,419,100	Pathogenic

Abbreviations: *BMP2*, bone morphogenetic Protein 2; FIX, coagulation Factor IX gene; *FVIII*, coagulation Factor VIII gene; HA, hemophilia A; IDA, iron deficiency anemia; IRIDA, iron‐refractory iron deficiency anemia; *LMAN1*, lectin, mannose binding 1; *TF*, transferrin; *TMPRSS6*, transmembrane serine Protease 6.

#### 3.7.2. HB

Unfortunately, there is a lack of studies performed on Saudi patients with HB. Only two studies were included in this review, as presented in Table 2.

#### 3.7.3. Combined Factors V and VIII Deficiency (F5F8D)

F5F8D is a rare genetic bleeding disorder; only one case was reported in KSA, as illustrated in Table 2.

### 3.8. Hereditary Hemochromatosis

A condition that interrupts iron homeostasis, causing systemic iron overload [38]. It is classified according to the genes involved into Type 1A (homozygote) (*HFE* gene C282Y), Type 1B (compound heterozygote) (*HFE* gene H63D), Type 1C (*HFE* gene S65C), Type 2 (juvenile hereditary hemochromatosis) (*HJV* and *HAMP* gene mutations), Type 3 (*TFR2* gene mutation), and Type 4 (*SLC40A1* gene mutation) [38].

One study involving 500 healthy individuals showed frequencies of 0.0% for C282Y and 14% for *H63D*, suggesting that in KSA, HFE hemochromatosis is predominantly associated with the *H63D* mutation [39]. A case also reported a 5‐year‐old boy diagnosed with autosomal recessive hereditary hemochromatosis, caused by a homozygous *HFE* gene variant *c.187C*>*G* (*p.His63Asp*) [40].

### 3.9. Iron Deficiency Anemia (IDA)

IDA is a significant health alarm in KSA, with studies reporting a prevalence ranging from 30% to 56% in the general population [41], especially among certain subgroups such as female adolescents aged 16–18 years (40.5%) [42], preschool children (20%–67%), school children (12.6%–50%), and pregnant women (22.7%–54%) [43]. Table 2 provides an overview of variants in *TF*, *TMPRSS6*, and *BMP2* genes that were linked with IDA in the Saudi population.

### 3.10. Leukemia

Leukemia is a group of malignancies that is triggered by hematopoietic stem cells and progenitor cells located inside the bone marrow. There are four types of leukemia, including acute myeloid leukemia (AML), chronic myeloid leukemia (CML), acute lymphoblastic leukemia (ALL), and chronic lymphocytic leukemia (CLL) [44]. According to national cancer registry data, leukemia ranks as the fifth most prevalent malignancy in KSA [45]. Another study revealed that among Saudi men, leukemia ranks as the third most widespread cancer, with ALL being the most common [46].

The x‐ray repair cross‐complementing Group 1 (*XRCC1*) binds to DNA repair‐related proteins and influences the base excision repair (BER) process. Multiple reports have suggested that *XRCC1*‐SNP *Arg399Gln* at Codon 399 is linked to cancer onset including leukemia [47]. A study identified 10 variants in the frequency of *XRCC1* Arg399Gln polymorphism in Saudis that differs significantly from the global populations [48]. Another study investigated the association of Ki‐ras2 Kirsten rat sarcoma viral oncogene homolog (*KRAS*) rs61764370T>G gene variation in 72 leukemia patients. The study defined a higher fraction of TT, GT, and GG genotypes in patients indicating that KRAS rs61764370GG genotype and G allele were linked with an amplified predisposition to leukemia among Saudis [49]. Several studies were performed on different types of leukemia, including AML, CML, ALL, and CLL patients to explore diverse gene association with the suitability to leukemia as revealed in Table 3.

**Table 3:** The described gene variants associated with the different types of leukemia, including AML, CML, ALL, and CLL in the Saudi population.

Source (ref)	No of cases	Gene	Mutations/variants reported (HGVS expression)	Protein change	dbSNP/ClinVar IDs	GRCh38 coordinates	Reported pathogenicity
**3.10.1 Acute myeloid leukemia (AML)**							
[49]	98 Saudi AML cases	*TIM-3*	NM_032782.3:c.574A>C	Intronic/splice effect predicted (no direct NP_ change)	rs10515746	Chr 5: 155,752,382	Increased susceptibility to AML
		*CTLA-4*	NM_005217.4:c.‐319A>G (5 ^′^ UTR/regulatory region)	Noncoding variant	rs231775	Chr 2: 203,865,038	Protective effect against AML
[51]	100 AML cases	*JAK2*	NM_005101.3:c.1849G>T	NP_005092.2:p.Val617Phe	rs387907409/VCV000021319.4	Chr 9: 5,082,143	Pathogenic
[51]	32 AML pediatric patients	*NPM1*	NM_002520.7:c.860_863dupTCTG (common insertion type A)	NP_002511.1:p.Trp288fs	VCV000109968.1	Chr 5: 170,839,418	Pathogenic
		*FLT3-ITD*	Internal tandem duplication (ITD) in Exon 14	In‐frame insertion in the juxtamembrane domain	VCV000213898.1	Chr 13: 28,044,000	Pathogenic
		*FLT3-TKD*	Tyrosine Kinase Domain (TKD) point mutation (e.g., D835Y)	p.Asp835Tyr	rs121913495/VCV000021406.1	Chr 13: 28,042,437 (for D835Y)	Pathogenic

[53]	100 AML patients	*KIR2DL1*	Gene presence/absence polymorphism	Inhibitory receptor	Not applicable	Chr 19: ~59.5 Mb region	Higher frequency in AML cases
		*KIR2DL5*	Gene presence/absence polymorphism (KIR2DL5A/B)	Inhibitory receptor	Not applicable	Chr 19: ~59.5 Mb region	Highest risk association with AML
		*KIR2DS2*	Gene presence/absence polymorphism	Activating receptor	Not applicable	Chr 19: ~59.5 Mb region	Increased risk of AML
		*KIR2DL3*	Gene presence/absence polymorphism	Inhibitory receptor	Not applicable	Chr 19: ~59.5 Mb region	Lower AML risk (protective)
		*KIR2DS4*	Gene presence/absence polymorphism (full‐length/truncated)	Activating receptor	Not applicable	Chr 19: ~59.5 Mb region	Lower AML risk (protective)
		*KIR2DL2*	Gene presence/absence polymorphism	Inhibitory receptor	Not applicable	Chr 19: ~59.5 Mb region	Lower AML risk (protective)

[54]	43 cases	*IDH1*	NM_002162.3:c.395G>A (R132H mutation)	NP_002153.2:p.Arg132His	rs11554137/VCV000025810.1	Chr 2: 205,820,387	Pathogenic (somatic driver)
			NM_002162.3:c.394C>T (R132C mutation)	NP_002153.2:p.Arg132Cys	rs121913499/VCV000025812.1	Chr 2: 205,820,386	Pathogenic (somatic driver)
			NM_002162.3:c.395G>C (R132S mutation)	NP_002153.2:p.Arg132Ser	rs121913500/VCV000025813.1	Chr 2: 2205,820,387	Pathogenic (somatic driver)
			NM_002162.3:c.315C>T	NP_002153.2:p.Gly105Gly	rs12720445	Chr 2: 205,823,281	Benign

[55]	100 AML	*MTHFR*	NM_005957.4:c.665C>T (alternative: c.677C>T)	NP_005948.3:p.Ala222Val	rs1801133/VCV000018596.10	Chr 1: 11,791,379	Benign (polymorphism)
			NM_005957.4:c.1286A>C (alternative: c.1298A>C)	NP_005948.3:p.Glu429Ala	rs1801131/VCV000018594.8	Chr 1:11,785,833	Benign
[56]	100 AML	*NAT2*	NM_000015.3:c.481C>T	NP_000006.2:p.Leu161Leu (synonymous)	rs1799929/VCV000021665.4	Chr 8: 18,393,212	Benign (polymorphism)
			NM_000015.3:c.857G>A	NP_000006.2:p.Gly286Glu	rs1799931/VCV000021667.4	Chr 8: 18,392,846	Benign
[57]	100 AML	*NQ01*	NM_000261.3:c.609C>T	NP_000252.1:p.Pro187Ser	rs1800566/VCV000014878.6	Chr 16: 50,064,288	Benign
			NM_000261.3:c.465C>T	NP_000252.1:p.Ala155Val	rs1131341/VCV000014879.4	Chr 16: 50,064,432	Benign
[57]	100 AML	*CYP1A1*	NM_000499.3:c.1384A>G	NP_000490.1:p.Ile462Val	rs1048943, VCV000021964.4	Chr 15: 77,589,709	Increased susceptibility to AML

**3.10.2 Chronic myeloid leukemia (CML)**							

[59]	22 cases	*IRF-1*	NM_002189.5:c.223A>G	NP_002180.1:p.Lys75Glu		Chr 5: ~135,765,800	Likely pathogenic
			NM_002189.5:c.664G>A	NP_002180.1:p.Glu222Lys		Chr 5: ~135,764,200	Likely pathogenic
			NM_002189.5:c.8985T>G (intronic variant)	Noncoding variant		1Chr 5: ~35,760,100	VUS
			NM_002189.5:c.8990T>G (intronic variant)	Noncoding variant		Chr 5: 135,760,100	VUS
			NM_002189.5:c.8995A>G (intronic variant)	Noncoding variant		Chr 5: ~135,760,100	VUS

**3.10.3 Acute lymphoblastic leukemia (ALL)**							

[60]	130 ALL cases	*BCR-ABL*	Reciprocal translocation t(9;22)(q34;q11)			Chr 9:q34 breakpoint and Chr 22:q11 breakpoint	Pathogenic
		*ETV6/RUNX1*	Reciprocal translocation t(12;21)(p13;q22)			Chr 12:p13 breakpoint and Chr 21:q22 breakpoint	Pathogenic (somatic driver in B‐ALL)
		*MLL-FOXO4*	Reciprocal translocation t(X11)(q13q23)			Chr X:q13 breakpoint and Chr 11:q23 breakpoint	Pathogenic (somatic driver in T‐ALL/AML**)**

[61]	20 samples	*TP53*	17p13.1 deletion (entire gene deleted)	Absent or nonfunctional protein product		Chr 17: 7,668,400–7,687,550 (entire gene locus)	Pathogenic
			NM_000546.6:c.524G>A	NP_000537.3:p.His175Arg	rs28934575/VCV000016462.11	Chr 17: 7,674,960	Pathogenic
[62].	68 cases		NM_000546.6:c.215G>C	NP_000537.3:p.Arg72Pro	rs1042522/VCV000016460.10	Chr 17: 7,676,561	Increased susceptibility to leukemia

[63]	150 ALL cases	*TLR3*	NM_003265.4:c.282C>T	NP_003256.2:p.Tyr94Tyr (synonymous)	rs5743312	Chr 4: 155,752,382	Increased ALL risk
			NM_003265.4:c.1234C>T	NP_003256.2:p.Leu412Phe	rs3775290	Chr 4: 155,739,788	Reduced ALL risk
			NM_003265.4:c.926C>A	NP_003256.2:p.Pro309His	rs3775296	Chr 4: 155,741,402	No significant association with ALL
			NM_003265.4:c.1234C>T	NP_003256.2:p.Leu412Phe	rs3775291	Chr 4: 155,739,788	

[64]	150 cases	*TLR4*	NM_138554.4:c.896A>G	NP_612564.2:p.Asp299Gly	rs4986790/VCV000021671.3	Chr 9: 120,490,568	Reduced ALL risk
			NM_138554.4:c.372C>T	NP_612564.2:p.Thr124Thr (synonymous)	rs1927906/VCV000219669.1	Chr 9: 120,491,192	Reduced ALL risk
[65]	165 ALL	*TNF–α*	NM_000629.4:c.‐308G>A (located in the 5 ^′^ UTR/promoter region)	Noncoding variant	rs1800629, VCV000018659.5	Chr 6: 31,575,567	**I**ncreased susceptibility to B‐cell ALL
[65]	136 ALL cases	*IL-17A*	NM_002190.3:c.124C>T (intronic/regulatory region relative to start)	Noncoding variant	rs3748067	Chr 6: 52,118,525	Reduced ALL risk
			Intronic/regulatory region (exact HGVS expression varies)	Noncoding variant	rs3819025	Chr 6: 52,118,251	No significant association with ALL risk
					rs8193036	Chr 6: 52,118,525	

**3.10.4 Chronic lymphocytic leukemia (CLL)**							

[67]	147 blood and bone marrow samples of B‐cell‐CLL cases		3q deletion (13q–)	Large structural variant (affects DLEU1, DLEU2, MIR15A, MIR16‐1 loci)	Not applicable	Chr 13: q14.2 region (~48 Mb)	Pathogenic
			Trisomy 12 (affects *MDM2* gene region)	Aneuploidy (extra copy of Chr 12)	Not applicable	Chr 12 (entire Chr)	
		*IGH*	gene abnormalities (*CCND1/IGH* translocation)	Translocation t(11;14)(q13;q32)	Not applicable	Chr 11:q13 breakpoint and Chr 14:q32 breakpoint	
		*ATM*	deletion (11q22.3 deletion)	Large structural variant (affects *ATM* gene locus)	Not applicable	Chr 11: q22.3 region (~108 Mb)	
		*P53*	deletion (*TP53* gene deletion, 17p13.1)	Large structural variant (affects *TP53* gene locus)	Not applicable	Chr 17: p13.1 region (~7.6 Mb)	

Abbreviations: B‐ALL, B‐cell lymphoblastic ALL; *CTLA-4*, cytotoxic T‐lymphocyte‐associated Protein 4; *CYP1A1*, cytochrome P450 Family 1 Subfamily A Member 1; *ETV6/RUNX1*, ETS variant transcription Factor 6/RUNX family transcription Factor 1; *FLT3*, FMS‐like tyrosine Kinase 3; *FLT3-ITD*, FMS‐like tyrosine Kinase 3‐internal tandem duplication; *FLT3-TKD*, FMS‐like tyrosine Kinase 3‐tyrosine kinase domain; *GST*, glutathione‐S‐transferase; GSTM1, glutathione S‐transferase mu 1; GSTP1, glutathione S‐transferase pi 1; GSTT1, glutathione S‐transferase theta 1; *IDH1/2*, isocitrate Dehydrogenases 1 and 2;*IL-17A*, interleukin 17A; *IRF-1*, interferon regulatory Factor 1; *JAK2*, Janus Kinase 2; *KIR2DL1*, killer cell immunoglobulin‐like receptor 2DL; *MLL-F0X04*, mixed‐lineage leukemia‐forkhead box O4 fusion gene () mutation was more frequently in T‐ALL; *MTHFR*, methylenetetrahydrofolate reductase; *MT-ATP6*, mitochondrially‐encoded ATP synthase membrane Subunit 6; *NAT2*, N‐acetyl Transferase 2; *NPM1*, Nucleophosmin 1; *NQ01*, NADPH quinone Dehydrogenase 1; *NR3C1*, glucocorticoid receptor; T‐ALL, T‐cell lymphoblastic ALL; *Tim-3*, T‐cell immunoglobulin mucin‐3; *TLR3*, Toll‐like receptor‐3; *TLR4*, Toll‐like receptor‐4; *TNF–α*, tumor necrosis factor‐alpha; *TP53*, tumor Protein 53; VUS, variant of uncertain significance.

### 3.11. Lymphoma

The aggressive lymphomas, which encompass Hodgkin lymphoma (HL) and non‐Hodgkin lymphoma (NHL), form a varied collection of illnesses that develop from a clonal expansion of lymphocytes [68]. According to the Saudi Cancer Registry (SCR), HL cases increased by 174.1%, whereas NHL cases rose by 80% between 2001 and 2020 in KSA [69]. Given this sharp rise, it is imperative to investigate genetic polymorphisms associated with lymphoma in the Saudi population.

#### 3.11.1. HL

Table 4 summarizes the genetic polymorphisms associated with HL in Saudi patients.

**Table 4:** Gene variants associated with HL in Saudi patients.

Source (ref)	No of cases	Gene	Mutations/variants reported (HGVS expression)	Protein change	dbSNP/ClinVar IDs	GRCh38 coordinates	Reported pathogenicity
[70])	100 tissue samples of CHL patients	*XPG*	NM_000124.3:c.3310G>C	NP_000115.2:p.Asp1104His	rs17655/VCV000109033.4	Chr 10: 102,873,436	Associated with overall survival (OS) in HL
		*CD163*	NM_001252988.2:c.681A>G	NP_001239917.1:p.Gln227Gln (synonymous)	rs75608120	Chr 12: 100,551,971	Associated with disease relapse (DR) in HL
[71]	100 CHL patients	*CD163*	NM_001252988.2:c.681A>G	NP_001239917.1:p.Gln227Gln (synonymous)	rs75608120	Chr 12: 100,551,971	Associated with disease relapse (DR) in HL
			N/A (promoter region)		rs61729	Chr 12: 100,554,435	Polymorphism; no significant association with disease relapse (DR) in HL
			N/A (promoter region)		rs11054197	Chr 12: 100,553,040	
			N/A (promoter region)		rs11054195	Chr 12: 100,553,059	
			NM_001252988.2:c.1172A>G		rs200642325	Chr 12: 100,551,480	
			NM_001252988.2:c.32G>A		rs61729510	Chr 12: 100,554,028	
			NM_001252988.2:c.1594+1G>A		rs150018775	Chr 12: 100,549,434	

[70]	100 patients	*BCL2*	NM_000633.2:c.‐938C>A (Promoter region variant)	Noncoding variant	rs2279115	Chr 18: 58,969,576	Polymorphism associated with increased lymphoma risk (G allele)
		*MCL1*	NM_005633.4:c.∗226T>G (3 ^′^ UTR variant)		rs9803935	Chr 1: ~151,363,400	

		*BIRC5*	NM_001168.3:c.919G>C (intronic variant, likely affects splicing)		rs17882312	Chr 17: ~63,607,450	Increased lymphoma risk (C allele)
		*BIRC5*	NM_001168.3:c.‐31G>C (promoter region variant)		rs9904341	Chr 17: ~63,607,150	Increased lymphoma risk (G allele)

[73]	61 HL	*MEGF11*	NM_080665.4:c.1054+193T>C (intronic)		rs150945752	Chr 15: ~45,690,400	Polymorphism associated with HL risk
		*CACNA1I*		NM_021659.3:c.5714+41C>G (intronic)	rs58055559	Chr 1: ~191,489,500	
		*DECR2*	NM_001007151.2:c.216C>T (synonymous)	NP_001007151.1:p.His72His	rs146760080	Chr 10: ~89,761,900	
		*STAB1*	NM_003144.3:c.1627+447G>A (intronic)	Noncoding variant	rs143894786	Chr 3: ~51,755,700	
		*ZNF526*	NM_001142517.2:c.491C>A (missense)	NP_001136000.1:p.Ala164Asp	rs144433879	Chr 19: ~53,607,900	
		*CPLANE1*	NM_001080517.2:c.2415+437C>T (intronic)	Noncoding variant	rs200612080	Chr 4: ~154,345,500	
		*DLK1*	NM_004092.3:c.∗1457T>A (3 ^′^ UTR)		rs105800	Chr 14: ~101,232,500	
		*RTN4RL2*	NM_001170701.1:c.2248+137T>C (intronic)		rs61745214	Chr 4: ~87,419,000	
		*PGRMC1*	NM_001206846.1:c.∗506G>A (3 ^′^ UTR)		rs145582672	Chr 1: ~150,001,800	

Abbreviations: *BCL2*, B‐cell lymphoma‐2; *BIRC5*, *survivin*; *CD163*, cluster of differentiation 163; *MCL1*, myeloid cell leukemia‐1; XPG, xeroderma pigmentosum complementation Group C.

#### 3.11.2. NHL

Ahmad et al. [74] tested 45 NHL Saudi patients for the *p53* mutation and identified a homozygous missense variant in Exon 5, c.536C>T (p.His179Tyr) in a single patient.

##### 3.11.2.1. Diffuse Large B‐Cell Lymphoma (DLBCL).

A study on 182 DLBCL Saudi patients revealed a substantial connection between the *GSTT1* null genotype, the *GSTP1* promoter variant *c.-2293C*>*T* (*rs1695, p.Ile105Val*) in the TT phenotype, and the *CYP1A12C* (*c.2455A*>*G, p.Ile462Val; rs1048943*) genotype with increased risk of DLBCL, whereas no effect was detected for the *GSTM1* null and *NQO1* genotype [75]. Also, O^6^‐methylguanine‐DNA methyltransferase (*MGMT*) promoter hypermethylation was observed in 71% of 100 DLBCL Saudi cases revealing that *MGMT* methylation is significantly associated with increased in the OS [76]. Another case–control study involving 160 DLBCL patients stated that patients carrying *MTHFR c.1286A*>*C* (*p.Glu429Ala*; rs1801131, 1298CC genotype) and the 1298C allele exhibit superior risk for DLBCL, respectively. Furthermore, combined genotypes *c.665C*>*C + c.1286C*>*C* (*MTHFR 677CC+1298CC*) and *c.665C*>*T + c.1286C*>*C (MTHFR 677CT+1298CC)* conferred an even higher risk of DLBCL [77].

### 3.12. Neutropenia

Neutropenia is a hematological condition characterized by absolute neutrophil count (ANC) of less than 0.5 × 10^9^/L [78]. The prevalence of neutropenia in Southern and Southwestern of KSA over a period of 5 years (2016–2020) ranged from 11% to 23% [79]. In a more recent study conducted between 2020 and 2022, the prevalence in Jeddah, Najran, and Asir regions was found to be 19.64%–35.2% [80], which reflects the high prevalence of this condition. Despite this high prevalence, genetic studies investigating the underlying causes remain limited. The genetic mutations associated with neutropenia in KSA are illustrated in Table 5

**Table 5:** Reported gene mutations associated with neutropenia in Saudi Arabia.

Source (ref)	No of cases	Gene	Mutations/variants reported (HGVS expression)	Protein change	dbSNP/ClinVar IDs	GRCh38 coordinates	Reported pathogenicity
[81]	One with congenital neutropenia	*HAX1*	NM_001011873.2:c.463_464insC	NP_001011873.1:p.Gln155Profs∗14 (p.Gln155ProfsX14)		Chr 1: ~151,807,170	Pathogenic
[82]	Five members of a single family	*G6PC3*	NM_138766.4:c.974T>G	NP_619658.2:p.Leu325Arg	rs770732899/VCV000184976.2	Chr 17: 40,899,944	
[83]	Two cases of Dursun syndrome		NM_138766.4:c.479C>T	NP_619658.2:p.Ser160Leu	rs767223746/VCV000529598.1	Chr 17: 40,900,439	
[84]	Two cases	*VPS45*	NM_001142416.1:c.1229T>C	NP_001136000.1:p.Leu410Pro	rs770146030/VCV000213155.1	Chr 1: ~151,807,170	

Abbreviations: *G6PC3*, glucose 6‐phosphatase catalytic subunit‐3; *HAX1*, HCLS1‐associated Protein X1; *VPS45*, vacuolar protein sorting‐associated Protein 45.

### 3.13. Sickle Cell Disease (SCD)

Although the incidence of SCD has declined in some regions of KSA, its overall prevalence remains among the highest globally. A systematic review by Ata et al. [4] reported that 51.3% of Arab SCD cases (22,708) were from KSA. The highest prevalence was in the Eastern Province (145/10,000), followed by the Southern, Western, and Central regions. The Saudi Premarital Screening Program estimated that 0.26% of adults are carriers, and 4.2% are affected. Despite its endemic status, national efforts to address SCD remain insufficient. The first documented cases in KSA were from the Eastern Province in the 1960s [85]. This high incidence led to the launch of multiple regional and national screening studies to assess the clinical characteristics and frequency of SCD genes in KSA.

Currently, there are four major sickle haplotypes related to a certain geographic region: “Senegal” (Atlantic West Africa), “Benin” (Central West Africa), “Bantu” (also called “CAR” for Central African Republic), and the Cameroon and “Arab–Indian” haplotype (also called the Asian or Saudi haplotype) found in India and parts of KSA [86].

Borgio, J. F. et al. [87] exposed a new *HBA2* gene conversion in cis or trans in 5.7% of 157 Saudi cases by direct sequencing of the *HBA2* and *HBA1* genes. This variant, termed the *α12* (*HBA12*) allele, results from a combination of *α*1 and *α*2 sequences. Three genotypes were detected: homozygous (‐*α*12 (3.7)/*α*1*α*12), heterozygous (*α*1*α*2/*α*1*α*12), and hemizygous (*α*1^-^(4.2)/*α*1*α*12) [87]. Table 6 summarizes the reported genetic mutations associated with Saudi SCD cases.

**Table 6:** Reported gene mutations associated with SCD Saudi patients.

Source (ref)	No of cases	Gene	Mutations/variants reported (HGVS expression)	Protein change	dbSNP/ClinVar IDs	GRCh38 coordinates	Reported Pathogenicgenicity
[88]	77	*HBB*	47 variants were identified across different coding segments. These included: one variant in Fragment 1, three in Fragment 2, two in Fragment 3, seven in Fragment 4, three substitutions in Fragment 5, two in Fragment 6, five in Fragment 7, seven substitutions in Fragment 8, two heterozygous substitutions in Fragment 9, three in Fragment 10, eight substitutions in Fragment 11, and four in Fragment 12.				
[89]	778		The dispersal of haplotypes for 746 Hb S (*HBB*: c.20A>T (p.Glu7Val; *Hb S*) homozygotes was as follows: 614 AI/AI, nine SEN/SEN (Senegal), 42 SEN/AI, nine CAM/CAM (Cameroon), one CAR (Central African Republic)/BEN (Benin), and 71 AI/atypical. In Hb S/*β*‐Thal (Hb S/*β*‐thal), the spread of Hb S haplotypes was as follows: 22 AI/AI, one CAM/CAM, four AI/SEN, and 5 AI/atypical				
[90]	69		LCR of the globin gene cluster was amplified, revealing 69 genetic variations, including 20 alterations in LCR‐HS1, six in HS2, multiple changes in HS3, four in HS5, eight in HS6, and 10 in HS7				
[91]	100		Five typical *β*‐globin haplotypes were identified. Benin, Bantu, and Senegal existed in homozygous state with 29%, 3%, and 1% frequencies, respectively. Remarkably, 29% of the cases presented atypical haplotypes in heterozygous state and 2% in homozygous state for the first time in the Jazan area				
[92]	630 with Arab–Indian	*ANTXR1*	NM_032228.4:c.118‐295C>G	Noncoding variant	rs4527238	Chr 2: ~187,015,100	Reduced HbF expression
			NM_032228.4:c.321A>G (synonymous variant)	NP_115599.2:p.Gln107Gln	rs35685045	Chr 2: ~187,015,300	
[91]	132 from Riyad City	*BCL11A*	NM_022884.3:c.192A>G	NP_073699.2:p.Gln64Gln (synonymous)	rs4671393	Chr 2: ~60,650,000	Increased HbF expression (GG/AG genotypes)
		*HBS1L-MYB*	Intergenic region	Noncoding variant	rs9399137	Chr 6: ~67,794,873	Increased HbF expression
			Intergenic region		rs28384513	Chr 6: ~67,794,873	
[94]	100 from northwestern of KS	*GSTT1*	*GSTT1* null genotype is considerably linked with amplified risk of SCD				

Abbreviations: *ANTXR1*, anthrax toxin Receptor 1; *BCL11A*, B‐cell lymphoma/leukemia 11A; GSTT1, glutathione S‐transferase Theta 1; *HBB*, hemoglobin subunit beta, HS3, hypersensitive site three of *HBB*; LCR, locus control region.

### 3.14. Thalassemia

KSA has the uppermost occurrence of thalassemia worldwide [95]; however, the percentage has declined substantially after the implementation of the premarital screening program in 2004 [96]. Alpha (*α*) thalassemia (*α*‐Thal) is an inherited condition marked by mutations in the *α*‐globin genes or their regulatory areas, causing a decreased or absent *α*‐chains, a critical part of hemoglobin. *α*‐Thal is highly prevalent in Jeddah (25%), followed by Makkah (23%), Taif (13.3%), and Al‐Ahassa (12.4%) [97], Southwestern Region, especially the Jazan Region (4.43%) [98]. On the other hand, beta‐thalassemias (*β*‐Thal) are triggered by decreased or lacking the beta‐globin part of hemoglobin [99]. Currently, *β*‐Thal prevalence is 13.6 per 1000 population in KSA [100].

Generally, *α*‐globin genes are of two types (*HBA2* and *HBA1*), whereas in KSA, a third type, which is *HBA12*, has been recognized in 5.7% of the Saudi population. The *HBA12* gene has the region starting −6 bp until 581 bp (3 ^′^ promoter, Exon 1, IVSI, Exons 2, and 5 ^′^IVSII) from the *HBA1* gene, and 774 bp (3 ^′^ enhancer) onwards from the *HBA2* gene. The region in‐between 581 bp and 774 bp (3 ^′^ IVSII, Exon 3 ^′^, and 5 ^′^ enhancer) was matching with *HBA1* and *HBA2;* hence, this region was considered as an indistinguishable region [87]. In terms of the Saudi population, there are three genotypes related to alpha‐globin genes, *α*_1_ *α*_2_/*α*_1_ *α*_2_, *α*_1_ *α*_2_/*α*_1_ *α*_12_, and *α*_1_ *α*_12_/*α*_1_ *α*_12_ [101]. The reported genetic mutations associated with thalassemia in KSA are illustrated in Table 7.

**Table 7:** Reported gene mutations associated with thalassemia in Saudi patients.

Source (ref)	No of cases	Gene	Mutations/variants reported (HGVS expression)	Protein change	dbSNP/ClinVar IDs	GRCh38 coordinates	Reported Pathogenicgenicity
[97]	625 samples	*HBA1/HBA2*	–*α*3.7 deletion (rightward deletion)	Absent/reduced alpha‐globin		~215 kb region	Pathogenic
		*HBA1/HBA2*	*α*2 IVS1(−5nt) mutation (c.115+2_115+6delTGAGG)	Splice site defect, reduced alpha‐globin	VCV000021385.1	~226.5 kb region	
		*HBA2*	*α*2 polyA‐1 (*α*2T.Saudi) variant (AATAAA>AAGAAA polyadenylation signal mutation)	Reduced mRNA stability, reduced alpha‐globin		~227.8 kb region	
		*HBA1/HBA2*	–MED deletion (Middle Eastern deletion)	Absent/reduced alpha‐globin		~215 kb region	
		*HBA1/HBA2*	–SEA deletion (Southeast Asian deletion)	Absent/reduced alpha‐globin		~215 kb region	
		*HBA2*	*α*2 polyA‐2 (*α*2T.Turkish) variant	Reduced mRNA stability, reduced alpha‐globin		~227.8 kb region	
		*HBA1/HBA2*	–*α*4.2 deletion (Leftward deletion)	Absent/reduced alpha‐globin		~215 kb region	
[102]	59 *β*‐Thal patients and carriers	*HBB*	NM_000518.5:c.118C>T	NP_000509.2:p.Gln40∗ (p.Gln39Ter)	rs114112001/VCV000005481.3	Chr 11: 5,227,100	
			NM_000518.5:c.315+1G>A (IVS‐II‐1 G>A)	NP_000509.2:p.? (splice site mutation)	rs113160021/VCV000005474.3	Chr 11: 5,227,300	
			NM_000518.5:c.92+1G>A (IVS‐I‐1G>A)	NP_000509.2:p.? (splice site mutation)	rs35355209/VCV000005470.1	Chr 11: 5,227,080	
			NM_000518.5:c.392G>C	NP_000509.2:p.Gly131Ala (p.Gly130Ala)	rs35875225/VCV000005463.3	Chr 11: 5,227,377	
[103]	140 *β*‐Thal patients	*HBB*	NM_000518.5:c.92+5G>C (IVS‐I‐5 G>C)	NP_000509.2:p.? (splice site mutation)	rs34765955/VCV000005473.2	Chr 11: 5,227,084	Pathogenic
			NM_000518.5:c.118C>T	NP_000509.2:p.Gln40∗ (p.Gln39Ter)	rs114112001/VCV000005481.3	Chr 11: 5,227,100	
	131 samples	*HBB*	NM_000518.5:c.410G>A	NP_000509.2:p.Gly137Ser		Chr 11: ~5,227,390	Likely pathogenic (novel missense)
			NM_000518.5:c.‐151C>T (promoter variant)	Noncoding variant		Chr 11: ~5,226,950	Likely pathogenic (novel promoter)
			NM_000518.5:c.68_74delAAGTTGG	NP_000509.2:p.Lys23fs		Chr 11: ~5,227,050	Pathogenic (novel frameshift)
			NM_000518.5:c.316‐3C>A (splice acceptor)	NP_000509.2:p.? (splice site mutation)		Chr 11: ~5,227,290	Pathogenic (Novel splice acceptor)
			NM_000518.5:c.‐31C>T (promoter variant)	Noncoding variant		Chr 11: ~5,226,950	Likely pathogenic (novel promoter)
[96]	25 *β*‐Thal patients from Taif City	*HBB*	NM_000518.5:c.92+5G>C (IVS‐I‐5 G>C)	NP_000509.2:p.? (splice site mutation)	rs34765955/VCV000005473.2	Chr 11: 5,227,084	Pathogenic
			NM_000518.5:c.27_28insG	NP_000509.2:p.Lys9Serfs∗19	rs35677157/VCV000005459.2	Chr 11: 5,227,039	
			NM_000518.5:c.132C>A	NP_000509.2:p.Lys44Asn	rs34388487/VCV000005483.2	Chr 11: 5,227,114	
[105]	66 *β*‐Thal patients	*HBB*	NM_000518.5:c.92+5G>C (IVS‐I‐5 G>C)	NP_000509.2:p.? (splice site mutation)	rs34765955/VCV000005473.2	Chr 11: 5,227,084	
			NM_000518.5:c.315+1G>A (IVS‐II‐1 G>A)	NP_000509.2:p.? (splice site mutation)	rs113160021/VCV000005474.3	Chr 11: 5,227,300	
			NM_000518.5:c.118C>T (CD39, p.Gln40∗)	NP_000509.2:p.Gln40∗	rs114112001/VCV000005481.3	Chr 11: 5,227,100	
			NM_000518.5:c.182_183del (IVS‐I‐3 ^′^end, 25 bp del)	NP_000509.2:p.? (splice site mutation)	rs35677028	Chr 11: 5,227,166	
			NM_000518.5:c.17_19del (CD5, p.Val6fs)	NP_000509.2:p.Val6fs	rs35004245	Chr 11: 5,227,034	
			NM_000518.5:c.92+6T>C (IVS‐I‐6 T>C)	NP_000509.2:p.? (splice site mutation)	rs35355210	Chr 11: 5,227,085	
			NM_000518.5:c.92+1G>A (IVS‐I‐1 G>A)	NP_000509.2:p.? (splice site mutation)	rs35355209/VCV000005470.1	Chr 11: 5,227,080	
			NM_000518.5:c.27_28insG (CD8/9+G)	NP_000509.2:p.Lys9Serfs∗19	rs35677157/VCV000005459.2	Chr 11: 5,227,039	
			NM_000518.5:c.135delC (CD44 ‐C)	NP_000509.2:p.Gly46fs	rs33959869/VCV000005484.1	Chr 11: 5,227,117	
Alwazani et al. [106]	100 *β*‐Thal cases	*HBB*	NM_000518.5:c.92+5G>C (IVS‐I‐5G>C)	NP_000509.2:p.? (splice site mutation)	rs34765955/VCV000005473.2	Chr 11: 5,227,084	Pathogenic
[107]	31 *β*‐Thal children	*HBB*	NM_000518.5:c.118C>T (CD39)	NP_000509.2:p.Gln40∗ (p.Gln39Ter)	rs114112001/VCV000005481.3	Chr 11: 5,227,100	
			NM_000518.5:c.92+1_92+25del (IVS‐I‐25 bp del)	NP_000509.2:p.? (splice site mutation)	rs35677028	Chr 11: 5,227,080 (deletion starts at +1)	
			NM_000518.5:c.315+1G>A (IVS‐II‐1 G>A)	NP_000509.2:p.? (splice site mutation)	rs113160021/VCV000005474.3	Chr 11: 5,227,300	
			NM_000518.5:c.27dupG (CD8/9 insertion)	NP_000509.2:p.Val10fs	rs35677157/VCV000005459.2	Chr 11: 5,227,039	
			NM_000518.5:c.92+5G>C (IVS‐I‐5 G>C)	NP_000509.2:p.? (splice site mutation)	rs34765955/VCV000005473.2	Chr 11: 5,227,084	
			NM_000518.5:c.25_26delAA (Turkish frameshift)	NP_000509.2:p.Lys9fs	rs35848529	Chr 11: ~5,227,035	
			NM_000518.5:c.132delC (Kurdish frameshift)	NP_000509.2:p.Lys44fs	rs33959869	Chr 11: ~5,227,114	
			NM_000518.5:c.92+5G>T (IVS‐I‐5 G>T)	NP_000509.2:p.? (splice site mutation)	rs115541577	Chr 11: 5,227,084	
[108].	Saudi family, consisting of parents and nine children	*HBB*	NM_000518.5:c.364G>C	NP_000509.2:p.Glu122Gln (p.Glu121Gln)	rs33930165/VCV000005494.4	Chr 11: 5,227,346	Pathogenic (Hb D‐Punjab)
			NM_000518.5:c.126_129delTTCT	NP_000509.2:p.Phe42fs	rs35848560/VCV000005482.2	Chr 11: 5,227,108	Pathogenic (beta‐thal frameshift)
			NM_000518.5:c.92A>G	NP_000509.2:p.Lys31Arg (p.Lys30Arg)	rs34960309/VCV000005480.3	Chr 11: 5,227,074	Pathogenic (beta‐thal missense)
[109]	166 Saudi patients with transfusion‐dependent *β*‐Thal	*HBA1* and *HBA2*	–*α*3.7 deletion (a 3700 bp rightward deletion)	Absent or reduced alpha‐globin protein production		Chr 16: The deletion breakpoints define a region approximately between 214,500 bp and 218,200 bp	Pathogenic (causes alpha‐thal)
[110]	104 transfusion‐dependent *β*‐thal patients in the Eastern Province of KSA	*HBA1/HBA2*	–*α*3.7 deletion (rightward deletion)	Absent/reduced alpha‐globin		Chr 16: ~215 kb region	Pathogenic (most frequent)
		*HBA1/HBA2*	–MED deletion (Middle Eastern deletion)	Absent/reduced alpha‐globin		Chr 16: ~215 kb region	Pathogenic (novel in KSA)
		*HBA1/HBA2*	–FIL deletion (Philippine deletion)	Absent/reduced alpha‐globin		Chr 16: ~215 kb region	Pathogenic (novel in KSA)
		*HBA1/HBA2*	–*α*20.5 deletion	Absent/reduced alpha‐globin		Chr 16: ~215 kb region	Pathogenic (Novel in KSA)
		*HBA1/HBA2*	*ααα* anti‐3.7 triplication	Increased alpha‐globin		Chr 16: ~215 kb region	Associated (Prognostic modifier)
		*HBA1*	NM_000558.4:c.43G>A (CD 14 nonsense)	NP_000549.1:p.Trp15∗ (p.Trp14Ter)	rs35817208	Chr 16: ~227,650	Pathogenic
		*HBA1*	NM_000558.4:c.178G > A (Hb Adana)	NP_000549.1:p.Gly60Asp	rs35677028	Chr 16: ~227,800	Pathogenic (Hb Adana variant)
		*HBA2:*	*NM_000517.4:c.92A>G* (polyA1 variant)	Noncoding (affects polyadenylation)	rs35486801	Chr 16: ~226,350	Pathogenic
		*HBA2*	NM_000517.4:c.428T>C (Hb Koya Dora)	NP_000508.1:p.Ter143Glnext∗20	rs35891361	Chr 16: ~226,100	Pathogenic (Hb Koya Dora variant)
		*HBA2*	NM_000517.4:c.178G>A (Hb Adana)	NP_000508.1:p.Gly60Asp	rs35677028	Chr 16: ~226,200	Pathogenic (Hb Adana variant)
		*HBA2*	NM_000517.4:c.2T>C (initiation codon loss)	NP_000508.1:p.Met1Thr (or p.0?)	None assigned	Chr 16: ~226,050	Pathogenic
[111]	174 transfusions dependent *β*‐Thal Saudi patient	*HBS1L-MYB*	rs9376090	Non‐coding variant	rs9376090	Chr 6: ~67,794,873 bp	Polymorphism associated with increased HbF
			rs9399137		rs9399137	Chr 6: ~67,794,873 bp	
			rs4895441		rs4895441	Chr 6: ~67,794,873 bp	
			rs9376090		rs9376090	Chr 6: ~67,794,873 bp	
			rs9402686		rs9402686	Chr 6: ~67,794,873 bp	
			rs9494142		rs9494142	Chr 6: ~67,794,873 bp	
[112]	four female transfusion‐dependent *β*‐thal patients	*ATRX*	NM_000489.5:c.623delA	NP_000480.2:p.Asn208fs		Chr X: ~76,827,000	VUS
			NM_000489.5:c.848T>C	NP_000480.2:p.Leu283Pro		Chr X: ~76,826,000	
			ATRX (intronic Variant 1)	Noncoding variant		Chr X: ~76.8 Mb region	
			ATRX (intronic Variant 2)	Noncoding variant		Chr X: ~76.8 Mb region	

### 3.15. Thrombophilia

Thrombophilia is a group of disorders characterized by an increased tendency of blood clotting [113]. Antithrombin (antithrombin III, AT), an important plasmatic inhibitor for activated coagulation factors, is encoded by the serpin Family C Member 1 (*SERPINC1*) gene [114]. A novel pathogenic homozygous mutation c.1320C>G (p.Phe440Leu) in *SERPINC1* was identified in a 7‐year‐old female patient [115].

Factor V deficiency (F5D) is characterized by varying symptoms, extending from asymptomatic to severe bleeding events [116]. A study involved 11 patients identified six sequence variations in the *F5* gene, including four missense mutations (*c.566C*>*T; p.Pro189Leu, c.6010*T>*C; p.Trp2004Arg, c.6443*T>*C; p.Met2148Thr, c.6604C*>*T; p.Arg2202Cys*), a deletion (*c.2613delA* (*p.Arg872Lysfs* ∗ *12*)), and a splicing variant (*c.1118+5G*>*T*) [117].

In another cohort of 108 patients, screening for the *F5*: c.1601G>A (p.Arg534Gln) allele demonstrated genotype frequencies of GG (89.8%), GA (8.3%), and AA (1.9%), confirming its association with increased venous thrombosis (VT) risk [118]. Another study recognized 19 variations in *FVL* gene. Formerly described variants in *F5*, *MTHFR*, *PROS1*, *PROC*, *F8*, *F9*, *SERPINA10*, *SERPIND1*, and *HRG* genes were characterized in 21 cases. One novel variant *c.356G*>*T* (*p.Gly119Val*) in the *F7* gene was recognized as pathogenic [119].

Also, among 1524 samples tested for *FVL* (*c.1601G*>*A; p.Arg534Gln*) and 1023 for prothrombin gene mutation (*FII*, *c.97G*>*A* (Prothrombin G20210A)) displayed 5.9% were positive for *FVL* (5.5% heterozygous and 0.39% homozygous), 2.9% of people were positive for *FII* (2.8% heterozygous and 0.1% homozygous) [120].

Additionally, two Saudi families with “Factor X (FX)‐Riyadh” have been recognized. A novel missense alteration in Exon 4 of the *FX* gene, *c.151G*>*A* (*p.Glu51Lys*) resulting in *Glu51Lys* replacement in the first epidermal growth factor‐like domain of *FX* was noticed [121]. Another case of congenital FX‐Riyadh in a young girl confirmed a homozygous pathogenic variant *c. 271G*>*A* (*pGlu91Lys*) [32]. Alike, a study identified the risk of ribosomal protein L5 (*RPL5*) gene variants and hepatic vein thrombosis (HVT) in Saudi cases and exposed that the rs182018447 (AA genotype) and rs559377519 (TT genotype) polymorphisms were significantly correlated with increased HVT risk [122].

### 3.16. Thrombocytopenia

Thrombocytopenia, defined as a platelet count of less than 150,000/*μ*L, is caused by diminished platelet production and/or amplified platelet loss. Table 8 display different variants in different genes associated with thrombocytopenia in Saudi patients.

**Table 8:** Different reported mutations associated with inherited thrombocytopenias in Saudi patients.

Source (ref)	No of cases	Gene	Mutations/variants reported (HGVS expression)	Protein change	dbSNP/ClinVar IDs	GRCh38 coordinates	Reported pathogenicity
[123]	1314	MPL	NM_005373.2:c.317C>T	NP_005364.1:p.Thr106Met	rs750046020, VCV000219665.2	Chr 1: 43,803,799	Pathogenic
[124]	6‐years‐old female	*SBF2 (MTMR13)*	NM_015112.5:c.659C>G	NP_056000.1:p.Thr220Arg		Chr 11: ~117,163,000	
[125]	Affected father–daughter	ANKRD26	NM_014878.3:c.473A>G	NP_055693.2:p.Asp158Gly		Chr 10: 114,818,460	
[126]	Two siblings with IBMFS	**MYSM1**	NM_001085487.2:c.1168G>T	NP_001078956.1:p.Glu390∗	rs748430752/VCV000859368.1	Chr 1: 156,666,400 (approx.)	
[127]	Saudi girl	THPO	Deletion between chr3:184088108‐184090520	Absent/nonfunctional protein	Not applicable (structural variant)	Chr 3: 184,088,108–184,090,520	
[128]	11‐month‐old girl	GNE	NM_005476.3:c.1732G>A	NP_005467.1:p.Gly578Ser	rs121918398/VCV000024976.2	Chr 9: 36,448,561	
[129]	2‐year‐old BCS	KIF15	NM_001008537.2:c.1501C>T	NP_001008537.2:p.Arg501∗		Chr 4: 93,820	
[130]	13‐year‐old THAMY	MPIG6B	NM_001007559.2:c.523C>T	NP_001007559.2:p.Arg175Cys	VCV000242571.1	Chr 1: 156,066,700	
[131]	14‐year‐old with MTP	DIAPH1	NM_005218.4:c.3633_3636delTTCT	NP_005209.2:p.Phe1212Aspfs∗21	VCV000109033.4	Chr 5: 149,697,600	
[132]	Five‐month‐old with cTTP	*ADAMTS13*	NM_139025.5:c.2882delC	NP_619551.3:p.Cys962Alafs∗3	rs1567119020/VCV000109033.4	Chr 9: 136,368,000	
[133]	A Saudi girl		NM_139025.5:c.1520G>A	NP_619551.3:p.Arg507Gln	rs121918454, VCV000003058.10	Chr 9: 136,375,000	

Abbreviations: *ADAMTS13*, ADAM metallopeptidase with thrombospondin Type 1 Motif 13; *ANKRD26*, ankyrin repeat domain containing 26; BCS, Braddock–Carey syndrome; cTTP, congenital thrombotic thrombocytopenic purpura; *DIAPH1*, diaphanous‐related Formin 1; *GNE*, glucosamine (UDP‐N‐Acetyl)‐2‐epimerase/N‐acetylmannosamine kinase; IBMFS, inherited bone marrow failure syndromes; *KIF15*, kinesin family Member 15; *MPIG6B*, megakaryocyte platelet inhibitor G6B; *MPL*, proto‐oncogene thrombopoietin receptor; MTP, macrothrompocytopenia; *MYSM1*, Myb‐like, SWIRM And MPN Domains 1; THAMY, thrombocytopenia, anemia, and myelofibrosis disorder; *THPO*, thrombopoietin.

### 3.17. Von Willebrand Disease (VWD)

VWD is caused by deficiencies or abnormalities in the VWF. VWD is categorized into three types: Types 1 (VWD1) and 3 (VWD3) are triggered by deficiency of VWF, and Type 2 (VWD2) is caused by production by defective VWF [134]. VWD prevalence in KSA is higher than in other populations, reaching 3.9% [135].

In 60 VWD1 cases, the sequencing of *VWF* Exon 18 revealed five variants. Three exonic missense substitutions (*c.2332G*>*A* (*p.Asp778Asn*)*, c.2365A*>*G* (*p.Thr789Ala*), *c.2344C*>*T* (*p.Arg782Cys*)), one synonymous substitution (*c.2385*T>*C* (*p.Ser795=*)), and two intronic variants (*c.2442+38G*>*T, c.2442+71G*>*C*) [136]. Another study in 100 healthy subjects demonstrated three unusual sequence variations (one missense; rs775479826, and two synonymous; rs1286572448 and rs369828268) in Exon 18 of the healthy people, compared with other recorded VWF variations included three missense substitutions (*c.2332G*>*A* [*p.Asp778Asn*], *c.2365A*>*G* (*p.Thr789Ala*), *c.2344C*>*T* (*p.Arg782Cys*)), and one synonymous (*c.2385T*>*C* (*p.Ser795*=)) [133]. Furthermore, the *VWF* SNP rs61748511 (*c.3445*T*>C* (*p.Cys1149Arg*)) was found to be significantly associated with increased risk of ischemic stroke in a cohort of 100 stroke patients, identifying the C allele as pathogenic [138].

Interestingly, VWD can also occur concurrently with other genetic blood disorders, such as hereditary spherocytosis (HS). However, the co‐occurrence of the two appears to be extremely rare. A case report of a 13‐year‐old boy diagnosed with both HS and Type 1 VWD presented a heterozygous variant in the *c.281G*>*A* (*p.Trp94* ∗) in the solute carrier Family 4 Member 1 (*SLC4A1*) gene, which led to a premature stop codon [139].

### 3.18. Wiskott–Aldrich Syndrome

WAS is caused by mutations in the WASP actin nucleation promoting factor (*WAS)* gene [140]. A study on a Saudi family with two WAS affected males revealed a novel splice donor‐site mutation *c.811+5G*>*C* in Intron 8 in a homozygous status causing the protein to have 68 missense codons and a premature stop [141].

## 4. Discussion

This review highlights the spectrum of gene mutations underlying IBDs in the Saudi population, providing critical insights into the local genetic landscape. High‐frequency disorders such as SCD, *β*‐thalassemia, G6PD deficiency, and GT remain strongly influenced by consanguinity and founder mutations, reflecting distinct regional and tribal genetic patterns. Specific variants in *ITGA2B* and *ITGB3* were linked to GT, whereas diverse mutations in *FVIII* and *FIX* genes underlie HA and HB, respectively. The *HBB* gene carries the highest number of pathogenic variants, with the *HbS* mutation, predominantly within the Arab–Indian haplotype, dominating SCD cases. Modifiers in *BCL11A*, *HBS1L-MYB*, and *ANTXR1* influence phenotypic severity, demonstrating the complex interplay between genetic and environmental factors. Likewise, the –*α*3.7 deletion remains the most prevalent *α*‐thalassemia variant in the Kingdom. Additional recurrent mutations were identified in thrombophilia‐associated genes (*F5*, *FII*, *MTHFR*, and *SERPINC1*), thrombocytopenia‐related genes (*MPL, ANKRD26, THPO, DIAPH1,* and *ADAMTS13*), and rare but significant variants in *WAS, FX*, and *F7*.

### 4.1. Trends and Gaps

Although the accumulated evidence reflects remarkable progress in characterizing genetic variants across diverse Saudi cohorts, there remains a lack of comprehensive, large‐scale genomic studies covering all regions. Many reports are hospital‐based and limited by small sample sizes, leading to incomplete representation of national mutation frequencies. Furthermore, data fragmentation and the absence of a unified variant‐reporting system hinder integration of genetic findings into public health practice.

### 4.2. Recommendations

To bridge these gaps, establishing a national mutation registry to centralize and harmonize genomic data across institutions is crucial. Such a registry would facilitate systematic documentation of pathogenic variants, enhance diagnostic precision, support cascade screening for at‐risk families, and guide preventive healthcare policies. Expanding premarital and newborn screening programs, together with equitable access to genetic counseling and diagnostic services across all provinces, is also crucial to achieving comprehensive national coverage.

### 4.3. Future Outlook and Vision 2030 Alignment

The growing knowledge of Saudi‐specific pathogenic variants provides an opportunity to advance precision medicine, aligning with the Vision 2030 Health Sector Transformation Program. By integrating genetic discovery into clinical and public health frameworks, the Kingdom can promote early disease detection, targeted therapeutic development, and improved healthcare equity. Sustained investment in genomic research infrastructure, interdisciplinary collaboration, and data‐sharing initiatives will be vital for translating these findings into clinical and societal benefit.

## 5. Conclusion

This review emphasizes the critical need for strengthening genomic research, national mutation registries, and equitable access to genetic testing in KSA. By integrating genetic insights into preventive healthcare and precision medicine, the Kingdom can significantly reduce the burden of IBDs. Moving forward, sustained investment in genomic infrastructure, multidisciplinary collaboration, and population‐based studies will be essential to fill existing knowledge gaps. These initiatives directly align with Saudi Vision 2030 of promoting innovation, advancing precision medicine, and improving the quality and accessibility of healthcare services nationwide.

## 6. Limitation

Although we made every effort to collect and include all relevant genetic blood disorders reported in the Saudi population, the review was inherently limited by the availability of published genomic or clinical data in peer‐reviewed sources. Consequently, some rare conditions or subtypes may have been omitted due to the absence of accessible or documented evidence at the time of data collection. Additionally, the review is subject to publication bias, as studies with novel or significant findings are more likely to be published. Many of the included studies were single‐center reports, often from urban tertiary hospitals, which may not reflect the broader genetic landscape or healthcare accessibility in rural areas. Moreover, the lack of meta‐analytic synthesis limits the ability to draw statistical comparisons. The review also applied language restrictions, excluding non‐English studies, and did not systematically include gray literature, which may have led to the omission of relevant but unpublished data.

## Author Contributions

All authors have made a significant contribution to the work reported.

## Funding

The authors extend their appreciation to the Deanship of Scientific Research, Vice Presidency for Graduate Studies and Scientific Research, King Faisal University, Saudi Arabia (Grant No. KFU241941) for funding.

## Disclosure

All authors have agreed on the journal to which the article has been submitted.

## Conflicts of Interest

The authors declare no conflicts of interest.

## Data Availability

Data will be made available on request.
